# Supplementary material for: Effect of Cephalosporin Treatment on the Microbiota and Antibiotic Resistance Genes in Feces of Dairy Cows with Clinical Mastitis
Source: Antibiotics (Basel). 2022 Jan 17;11(1):117. doi: 10.3390/antibiotics11010117 (PMC8773067; doi:10.3390/antibiotics11010117)
Supplement: Supplementary file 1 [file antibiotics-11-00117-s001.zip › antibiotics-1529069-SI.pdf]

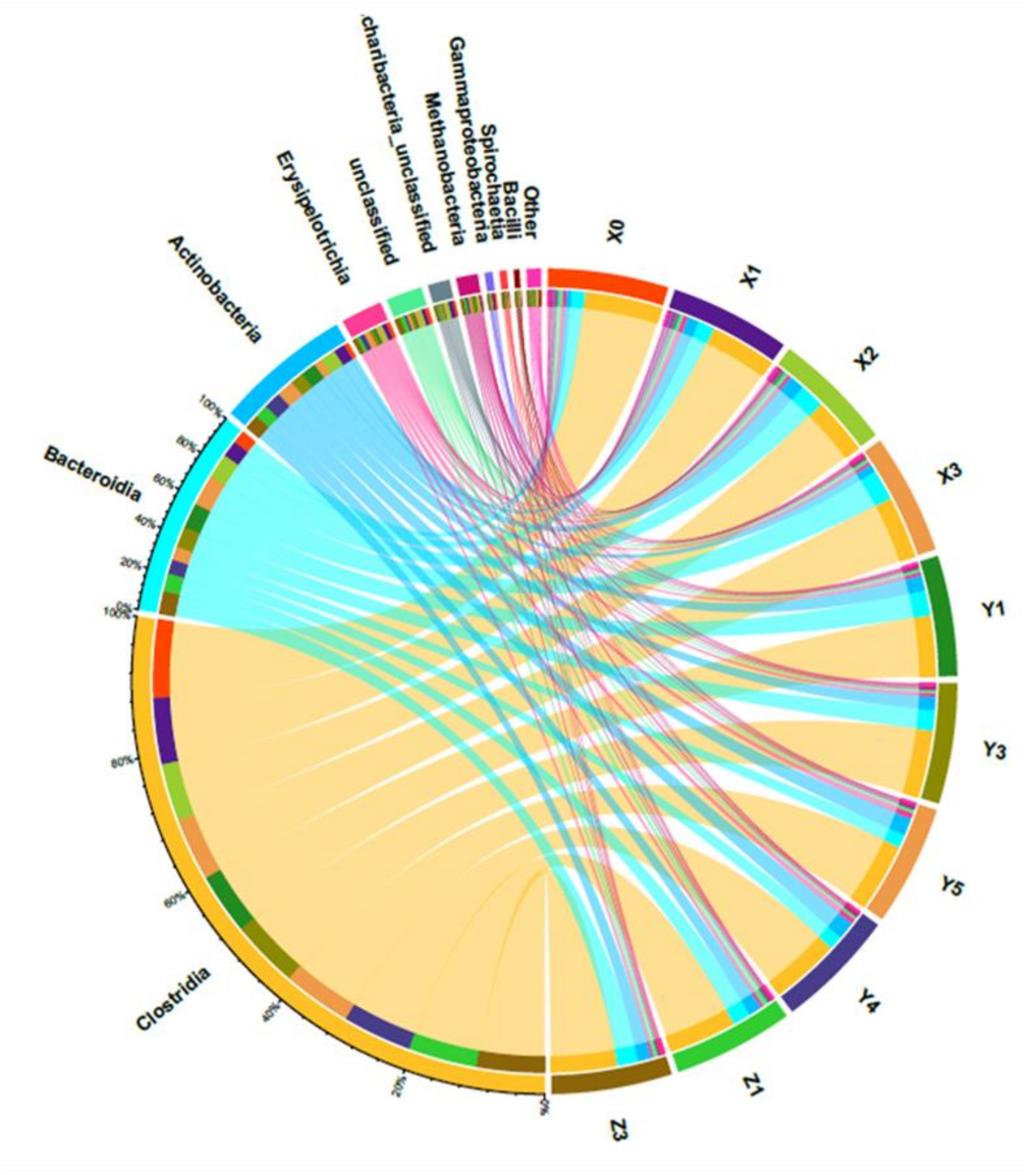

Figure S1 Microbiota composition at the class level samples from different periods. The top four family were descripted for each period, and all other family were grouped for "Other" or "Unclassified". X0, X, Y, and Z indicates the periods of day 0, medication, withdrawal, and recovery.

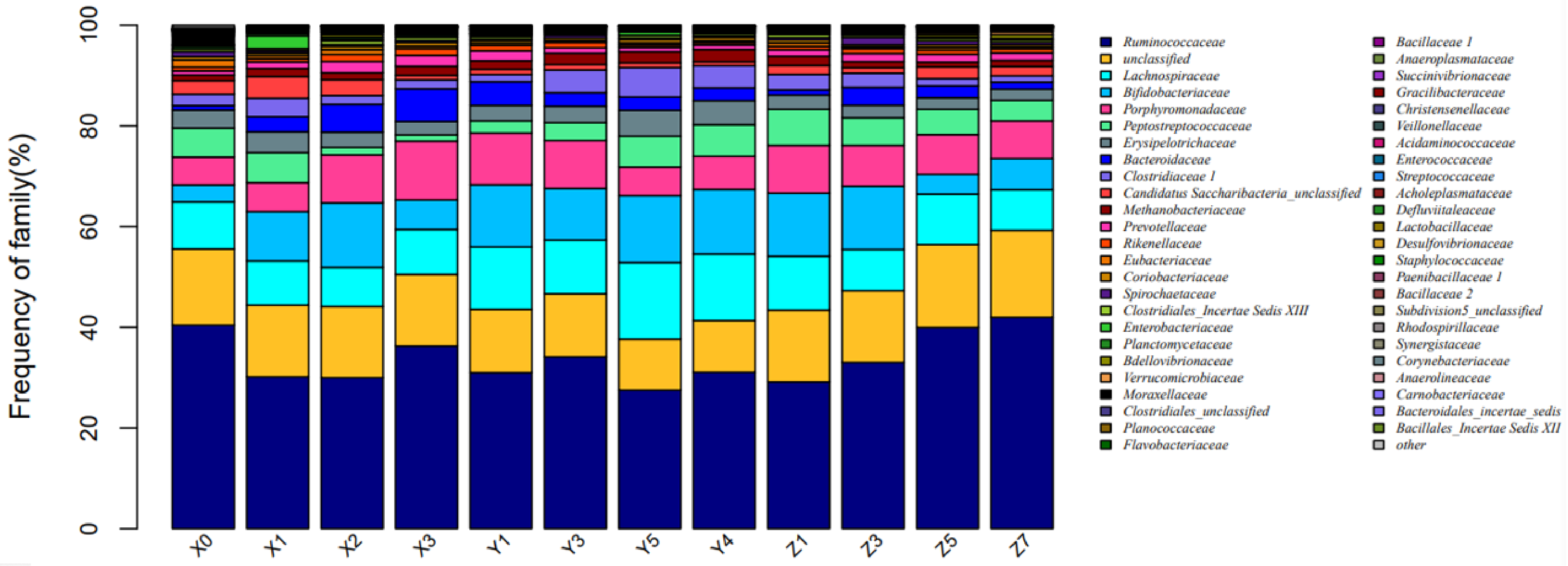

Figure S2 Microbiota composition at the family level samples from different periods. The top four family were descripted for each period, and all other family were grouped for “Other” or “Unclassified”. X0, X, Y, and Z indicates the periods of day 0, medication, withdrawal, and recovery.

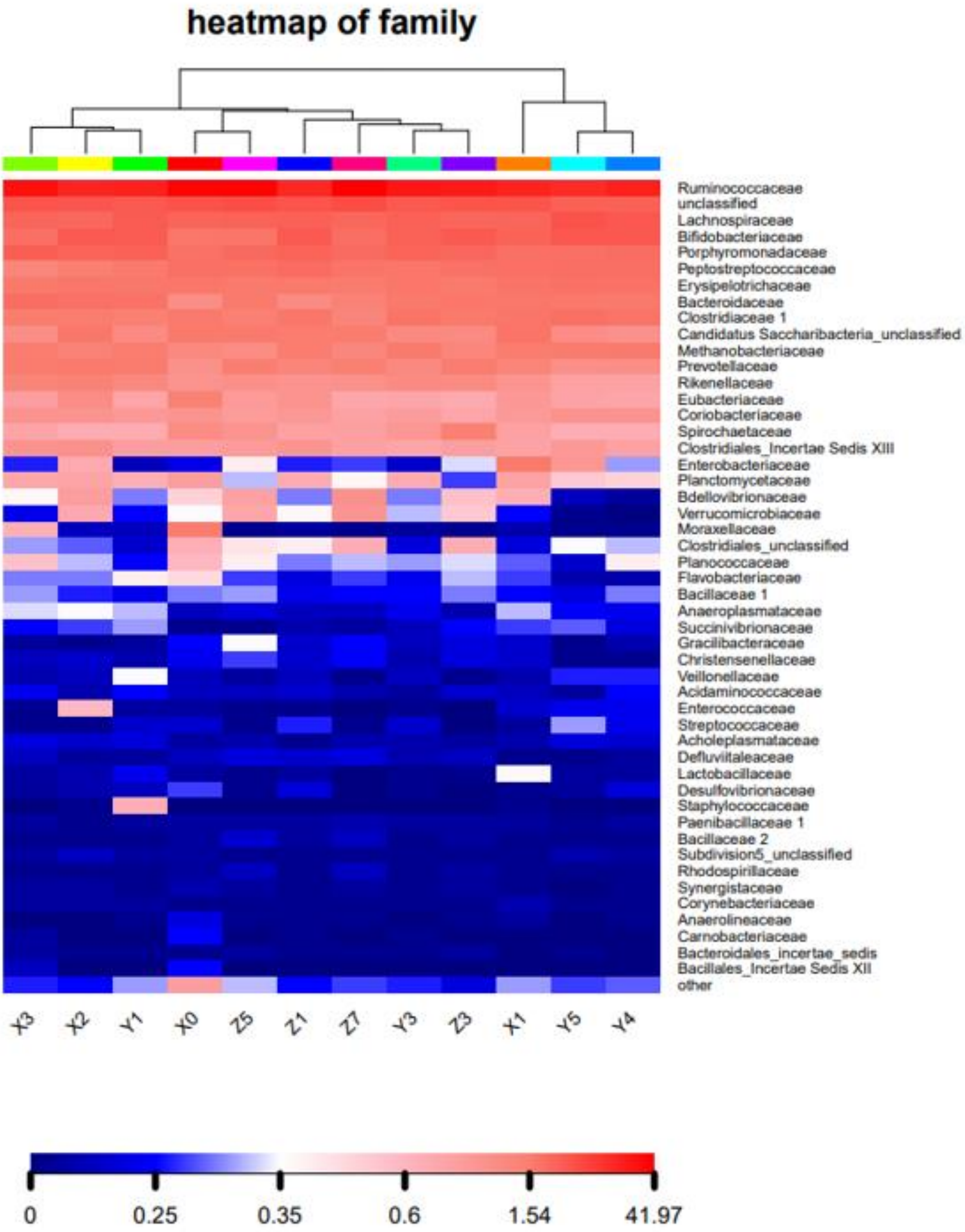

Figure S3 Alterations of bacterial genera abundance in the feces at different periods, heatmap showing the microbiota profile in the different periods, the blue color represents lower abundance of the microbiota and red color indicates the higher abundance.
